# Can wastewater monitoring protect public health in schools?

**DOI:** 10.1016/j.lana.2023.100475

**Published:** 2023-03-16

**Authors:** Francis Hassard, Suniti Singh, Frédéric Coulon, Zhugen Yang

**Affiliations:** Cranfield University, School of Water, Energy and Environment, Cranfield, MK43 0AL, UK

Schools are high-risk environments for infectious disease transmission, e.g., influenza, invasive group A streptococci infection, and rotavirus gastroenteritis affecting school-age children and potentially spreading to households.^1^ Throughout the pandemic, COVID-19 disease was generally mild for children, but led to significant social disruption due to school closures, impacting education, wellbeing and mental health and socioeconomics. Hence, timely detection and early warning of infections is crucial to prevent the spread of outbreaks. Wastewater-based surveillance (WBS) is a growing approach reliant on identification and quantification of wastewater biomarkers (e.g., gene fragments), transforming wastewater into a repository of data about population health. WBS is aggregate, anonymous, sensitive, and cost effective^2^ and can be used to assess infection burden and detect emerging variants in near real-time.^3^

In this issue Fielding-Miller et al.^4^ report on a pilot study (safer at school early alert) using WBS and surface swab testing in nine US school sites during the Alpha wave of COVID-19 when vaccine protection was not widespread. The study found that both wastewater and surface swab sampling could provide aggregate and anonymous data on infection trend within schools, with 93% of COVID-19 cases detected in wastewater. Diagnostic testing rates also improved throughout the study culminating in 99% for staff and 60% for students. Overall uptake of testing increased within study schools (78%) and was higher than similar schools not participating in the study (13%), suggesting that the data generated increased positive behaviours towards health protection in schools. Reactive effects of these experiments at the schools probably increased local testing compliance, therefore it is not clear whether this aspect of the study can be translated to other schools. The authors showed elegantly that resolving individual infections from environmental samples is possible using RNA sequencing and phylogenetic characterisation of variants. The authors estimate the cost of WBS in schools to be $42–120 per student per year (based on 300–850 pupils respectively), making it a promising approach for congregate settings with elevated disease risks (hospitals, care homes etc).^5^ Although economies of scale could reduce this cost considerably, at current costs monitoring a whole school using wastewater monitoring approximately 0.8-to-two times cheaper than the cost of a single diagnostic test undertaken in the US. If the approach was implemented over a school of 300 pupils the authors estimate the cost would be 250-fold less overall than diagnostic testing the school with equivalent accuracy to detect incidence (numerator) and school population coverage (denominator).

Petros et al.^6^ further demonstrated the value of collecting social data including Wi-Fi colocation information alongside WBS data, to predict COVID-19 infection risk in university students. Lengthy social interactions and contact sports identified as key risk factors. This work has important implications for outbreak prevention schemes in schools as activities likely to lead to more infections (e.g., contact sports etc.) could be scaled during periods of heightened risk. WBS has been further extended to monitor other diseases such as influenza and polio in the UK and US. For example, Vo et al.^7^ conducted a community-level wastewater monitoring programme to detect influenza viruses in elementary schools and identified vaccine-resistant influenza strains in this vulnerable population, coinciding with periods of enhanced risk such as easing of controls. However, it remains unclear how sensitive and responsive the monitoring system needs to be to mitigate or prevent an outbreak, particularly in schools. Additionally, in low- and middle-income countries or remote rural settings without established sewage networks, and in high schools where students transit between classrooms and sites, it may be difficult to establish links between viral load and cases. In some low-income settings (Belize and Guyana) access to basic sanitation services in primary schools is greater than equivalent household connections.^8^ In such scenarios, WBS in schools could enable monitoring infectious diseases in these communities. Many challenges remain for implementation of WBS in schools in the Global South, but it should be considered a priority as conventional levers for public health protection such as availability of vaccines and diagnostic testing are absent.^9^

The results presented by Fielding-Miller et al.^4^ suggest WBS could be implemented into a tiered surveillance approach for monitoring infectious disease which includes i) WBS for population-based testing which is representative and ii) genomics to efficiently monitor disease trends and mutations that are increasing in frequency or have been categorized as variants of concern.^10^ A framework for outbreak response could allow for the timely identification of risk factors, proximity and location patterns,^6^^,^^11^ and integration of multiple data sets to formulate a robust early warning system for public health in schools and beyond.^6^ Can wastewater monitoring help protect public health in schools? – yes! Do children defecate at school? – it depends on the child …

## Contributors

FH drafted the original manuscript, SS, FC and ZY reviewed and commented on manuscript.

## Declaration of interests

We declare no competing interests.
